# Associations of internet access with social integration, wellbeing and physical activity among adults in deprived communities: evidence from a household survey

**DOI:** 10.1186/s12889-019-7199-x

**Published:** 2019-07-02

**Authors:** Ade Kearns, Elise Whitley

**Affiliations:** 1 Urban Studies, School of Social & Political Sciences, University of Glasgow, 25 Bute Gardens, Glasgow, G12 8RS UK; 2 MRC/CSO Social and Public Health Sciences Unit, University of Glasgow, 200 Renfield Street, Glasgow, UK

**Keywords:** Internet access, Deprived communities, Loneliness, Wellbeing, Older people

## Abstract

**Background:**

There are arguments for and against the wellbeing effects of internet use, with evidence shifting from negative to positive over time, although the effects are partly dependent upon the population sub-group concerned. There are good grounds for anticipating that the internet could be beneficial to people living in deprived communities, but this group has rarely been studied.

**Methods:**

Data are from a cross-sectional, face-to-face survey of adult householders (*n* = 3804) in 15 deprived communities in Glasgow, UK. Respondents were asked whether they used the internet and, if so, how they usually accessed it: at home, via a mobile phone, in a public venue, or other means. Data were also collected on social contact and support, use of amenities, sense of community, wellbeing, loneliness, and physical activity.

**Results:**

There were inequalities in internet access within deprived communities, with use of the internet lowest among older people, those with a long-standing illness, and those with no educational qualifications. Some social benefits were associated with internet access, such as frequency of contact with neighbours, available financial social support, and greater use of social amenities and shops. Internet users were also less likely to report feeling lonely and had higher mental wellbeing scores. Respondents who used the internet were also more physically active. However, community cohesion and empowerment variables were very similar among internet users and non-users. Several of the positive associations with internet access were more marked for those who accessed the internet at home and for older people. These are new findings in respect of deprived communities.

**Conclusions:**

Extending internet access for people in deprived communities is worthy of further consideration in the context of government objectives for tackling social isolation and increasing wellbeing. The results also suggest that greater digitisation of public services may not result in greater cohesion and empowerment in deprived communities, as is often assumed, but rather has the potential to reinforce social inequalities.

## Background

The pros and cons of internet use have been debated for years, with many studies focusing on young people and reporting mainly negative associations. Early concerns that internet use served to reduce people’s social circles and increase the incidence of loneliness and depression [1, 2] have been joined by later studies reporting apparently beneficial effects for depression from the social links made or maintained via the internet [3]. This difference over time has been ascribed to the advent of social networking sites and the changing ways in which people use the internet [4, 5]. Although studies have investigated particular population sub-groups such as children and adolescents [6–9], college students [10–12] and older people [13, 14], few studies have explored internet use in deprived communities. This is an important omission as the rate of internet access in such communities is low, inequalities are likely to be high, and the scope for potential benefits great.

### Negative effects of internet use

Existing evidence on the negative effects of internet use can be considered in three broad categories: lower social connections; negative mental health effects; and lower levels of physical activity. Greater use of the internet has been associated with declines in intra-family communication, reductions in social networks, and a general reduction in social engagement [2, 15]. Two explanations for these effects have been suggested [2]: that internet use, being private and time consuming, displaces social activity leading to social withdrawal and that the internet displaces strong ties, with on-line relationships tending to be less tangible, less available and less sensitive to a person’s situation than face-to-face friendships. Moreover, the number of friendships made on-line fails to counteract the loss of physical, proximate friendships as a result of spending time on the internet.

Many studies have reported negative effects of internet use on mental wellbeing [16–18] and increased risk of depression [19]. A number of reasons for these effects have been suggested. Poor or declining mental health are seen partly as a product of the social isolation caused by internet use, and consequent feelings of loneliness [2, 20]. However, this line of argument has been contested through evidence that loneliness masks the effects of social anxiety and that neurotic personalities, particularly women, who are lonely endeavour to use the internet as an alternative way of forming social relationships [21]. A further major pathway to lower mental wellbeing is upward social comparison and associated envy. This is made more possible by social networking sites that enable people to present particularly positive images to similar people who devalue their own lives or self-presentation in comparison [4, 22, 23]. Studies have shown that those disposed to making comparisons of themselves with others have lower self-perceptions after internet use [24, 25], and that the negative relationship between social network site useage and subjective wellbeing is mediated by envy [16, 26]. Internet use can also raise anxieties about issues covered repeatedly by news reports and debates, e.g. terrorism; climate change; natural disasters; knife crime, or result in other negative outcomes for those who are socially anxious. Paradoxically, social anxiety can produce a preference for on-line social interaction and, indirectly, cause negative outcomes or ‘problematic internet use’ [27], defined as over-use with infringement upon other social activities. Specific problematic internet use also includes: being both a perpetrator and a victim of ‘trolling’, whereby there is a deliberate attempt to enrage people or cause disputes on the internet; computer rage, when users become frustrated with their computer or the internet; and internet addiction, where people become overly-dependent on the internet [28].

Lastly, internet use has been associated with lower levels of physical activity, longer periods of sitting and higher incidence of overweight and obesity, although these are most often studied in adolescents [29–32]. Among adults, internet use has been associated with overweight and obesity [33] and low levels of physical activity [34]. However, the relationship may not be uniform for all types of physical activity, with one study reporting general internet use to be positively associated with strenuous activity, but use of the internet for gaming to be negatively associated with physical exercise that strengthens muscles [35].

### Positive effects of internet use

The effects of internet use are dependent on the nature of the internet use, and several key distinctions can be made. The first distinction is between *capital-enhancing uses* and *recreational uses* of the internet, the former referring to the use of the internet to access or create opportunities and resources (e.g. for work, career, status) and the latter to transitory consumption and entertainment via the internet [36, 37]. Clark et al. make a more subtle, qualitative distinction in the way people use the internet, arguing that the effects of social network sites depend on the user’s ‘interpersonal-connection behaviours’ [5]. Verduyn et al. also argue that wellbeing outcomes from internet use depend upon whether there is *active or passive usage* [23]. Human-capital and social-capital uses of the internet are viewed as beneficial for social inclusion, advancing an individual’s knowledge and participation [35, 38]. In terms of connection behaviours, those that advance the human desire for acceptance and belonging are seen as being good for wellbeing, and behaviours that are ‘non-connection-promoting’ are seen as detrimental [5]. Active internet usage, which includes ‘activities that facilitate direct exchanges with others’ and which often produces information, is considered to be beneficial. In contrast, passive internet usage, which involves consuming information from others without direct communication or exchange with them, is considered to result in many of the negative consequences of internet use [23].

Reported advantages of the internet and social network sites are lower costs of maintaining relationships that might otherwise fade, and the ability to activate latent friendships to create stronger ties [23, 39]. Active internet use can increase both bonding and bridging social capital, greater feelings of social support and lower levels of loneliness [40]. On top of these general gains, certain types of people, most notably those who have social phobias such as a fear of being observed, or who are socially anxious, can benefit from feeling safer, more confident and more in control on the internet [41, 42]. Moreover, the relief of social anxiety online may lead to reduced anxiety offline in due course [43]. For those with mental health problems, the internet has been described as empowering in a number of ways: searching for information about symptoms and forms of treatment; finding others in similar circumstances who offer mutual support; publishing personal accounts and user perspectives of mental health services as a form of therapy and validation [44]. However, these benefits of internet use assume that those with mental health problems are capable of engaging positively with the internet, have access to it (not necessarily true for those in institutions), and are not otherwise prevented by other disabilities or low incomes.

### Deprived communities

There are good reasons for investigating patterns and associations of internet access in deprived communities, although this has not been done very often. Issues of inequality, or unequal access to the internet, i.e. the so-called first-level ‘digital divide’ [45], are likely to exist within such communities, particularly between those in and out of work, and between those who own or rent their homes. In the case of housing tenure, those in rented housing may be less likely to have internet access unless landlords provide internet services to tenants as part of the tenancy agreement, unlike owner occupiers for whom internet network services may be seen as part of the asset of the home. Issues of disadvantage may also be apparent whereby poor skills, faulty equipment, and poor network connections undermine the quality of internet access, something shown to impact the potential health gains from internet use [46].

Conversely, the circumstances of deprived communities are such that internet access and use could have particularly positive impacts. Worklessness and inactivity are significant issues in deprived communities, along with greater social isolation that goes with a lack of regular involvement in work outside the home. The internet may provide a means to overcome these things by providing ‘information about educational, career and community participation opportunities’ [47]. The fact that many people are on low incomes means that the internet may be valuable in providing a means to purchase goods more cheaply than in shops, and in enabling the maintenance of social relationships and development of social capital at lower cost than regular face-to-face meetings would require [23, 48]. On-line communication and identity can also enable people to avoid the effects of area-based prejudice by creating an identity free of place identifiers when connecting with others. Internet-based groups can also be a way for communities to create their own stories and history to counter the stigmatising discourse that often dominates media coverage of poor communities [49]. In addition the internet may assist people in deprived communities in providing sources of solidarity and support to help them cope with problems of poverty through sourcing practical and emotional advice and assistance, with participation in social network sites shown to mitigate inequalities in social support availability [50].

In one of the few studies of internet access and wellbeing in deprived communities, conducted in London [47], internet access at home was associated with higher levels of hope and happiness and lower levels of mental disorder symptoms. Those with internet access were also more socially connected with friends and family (though not neighbours) and reported higher levels of emotional and financial social support, with social connections and social support mediating the relationship between internet access and wellbeing. However, those with internet access were also more sedentary, spending more time sitting each day. Inequalities were reported within the deprived communities, with those with internet access being younger, more educated, and more likely to be employed or in full-time education. The relationship between internet access and wellbeing was found to be ‘marginally stronger’ for males, those with lower incomes and lower education, and immigrants; no differences in the association were found by age.

### Research aim

We add to existing evidence on the potential effects of internet access in deprived communities using a study of deprived communities in a different city and region of the UK, and, in contrast to the London study, we distinguish between internet access via mobile phone or other means (predominantly access via home computer); selectivity in means of internet access may be an important consideration for those living in deprived communities where internet access and incomes are relatively low. Our aim is to understand whether internet access, via different means, is associated with social, wellbeing and activity outcomes in deprived communities in Glasgow. Our focus is on social contact and support, use of amenities, sense of community, loneliness and wellbeing. We also specifically explore associations with internet use for those aged 65+, as this group is often ignored in studies of the effects of internet access, generally because use is assumed to be low and irreversible. However, increasingly older populations may have a lot to gain from using the internet, for example in combating cognitive decline and social isolation.

## Methods

### Context and data

The study was conducted in Glasgow, one of the most deprived cities in the UK: 46% of the city’s population live in neighbourhoods classified as the most deprived in Scotland, with the rate of workless households, at 15%, twice the national average [51]. The city has a large social rented housing sector, making up 39% of the housing stock, over three times the national rate. Internet access costs are an important consideration for low income households living in deprived communities, and there are a wide variety of deals available. Typical home broadband costs in the city range from £17–£35 for a slow minimum speed connection (10 Mb) and £21–£40 per month for a medium speed connection (30 Mb) and a 12 month contract [52]. The cost of a typical 2 year plan to purchase a mobile phone with 64 GB capacity and an internet allowance of 5GB of data per month ranges from around £23–£46 per month, although there are deals at below £20 with less internet allowance [53]. In both cases, there may also be upfront one-off costs in addition, to aid the purchase of either a tablet/laptop or a mobile phone.

This research was conducted in 15 deprived areas across Glasgow, six of which were subject to ongoing area regeneration and all having a social housing share above the city rate. The study uses data from a 2015 household survey carried out as part of an examination of the health and wellbeing impacts of regeneration [54]. A random selection of addresses from the postal address file were selected in nine of the study areas, with all dwellings included in the six regeneration areas. The study size was established in order to have 80% power of detecting a 5% reduction in the prevalence of common conditions (e.g. psychological symptoms or respiratory difficulties) in the main groupings of study areas (regeneration areas and non-regeneration areas) [54]. Respondents were householders or their partners living in any housing tenure within the study areas. The survey asked questions about housing, neighbourhoods, communities, health and wellbeing and household employment and finances, with one adult householder interviewed per household. A copy of the survey questionnaire is available online [55]. The survey achieved a response rate of 47.0% and a total of 3833 completed interviews. The main reasons for non-participation were non-response (53% of non-participant addresses), refusals (42%) and language difficulties or unavailability (5%). The data were weighted by age, sex and housing tenure to reflect the known characteristics of the study areas, and by study area population size within the total sample.

### Measures

#### Internet access

Respondents were asked how they usually accessed the internet for their own use, with 11 categories of multiple response, as well as allowing people to say they did not use the internet. The latter did not differentiate between those for whom access to the internet was difficult (e.g. due to cost) and those for whom non-use might be a choice (e.g. older people unfamiliar with, or who see no need for, the internet). From these responses we classified people as (1) non-users of the internet, (2) users of the internet by mobile phone only, (3) users of the internet by mobile phone and other means (including via a computer/laptop/tablet at home, and/or outside the home via internet café, computer at work, public library, public wifi, school or college, and landlord’s offices), and (4) users of the internet by other means only (i.e. any means other than mobile phone, including at home or elsewhere).

#### Social contact and support

Respondents were asked how often they had social contact in three forms: meeting up with relatives; meeting up with friends, and speaking to neighbours. From the responses, we identified those people who had each of these forms of contact at least weekly, i.e. ‘one a week or more’ or ‘most days’. For social support, the survey asked respondents how many people, not including those they live with, they could ask for different kinds of help: to go to the shops if they were unwell; to lend them money to see them through the next few days; and to give them advice and support in a crisis. The response categories (‘don’t know’ or ‘wouldn’t ask’, ‘none, ‘one or two’ or ‘more than two’) were combined to identify those people who had at least one person who could provide each of the forms of social support – practical, financial and emotional.

#### Use of amenities

The survey inquired about the use of 11 amenities in the previous 7 days. We divided these into two groups and identified those people who had used social amenities (covering sports and leisure, social venues, library, community centre and place of worship) and those who had used shops (including post office, local grocers, supermarket, shopping centre).

#### Sense of community

Community questions covered familiarity and belonging, trust and reliance, and empowerment. Respondents were asked to describe how many of the people in their neighbourhood (defined as an area within 5–10 min walk of their home) they knew. We compare those who said they knew ‘most’ or ‘many’ people in the area versus ‘some’, ‘very few’ or ‘no-one’. Respondents were also asked to what extent they felt they belonged to the neighbourhood and to what extent their neighbourhood was a place where neighbours looked out for each other; in both cases, we examine those who answered ‘a great deal’ versus ‘a fair amount’, ‘not very much’ or ‘not at all’. Respondents were presented with two statements about trust and reliance upon others in the area. The first statement concerned informal social control (‘It is likely that someone would intervene if a group of youths were harassing someone in the local area’) and the second concerned perceived honesty (‘Someone who lost a purse or wallet around here would be likely to have it returned without anything missing’). In both cases, we considered those who ‘strongly agreed’ or ‘agreed’ with the statements versus those who were neutral or disagreed. Lastly, respondents were given three statements about empowerment: one about influence (‘On your own or with others you can influence decisions affecting your local area’); one about proactivity (‘People in this area are able to find ways to improve things around here when they want to’); and one about service responsiveness (‘The providers of local services, like the council and others, respond to the views of local people’). We considered those who ‘strongly agreed’ or ‘agreed’ with these statements versus those who were neutral or disagreed.

#### Wellbeing

We used two wellbeing measures. Loneliness was assessed by asking respondents how often they had felt lonely over the last 2 weeks: all of the time, often, some of the time, rarely or never. This is similar to a question used by the Mental Health Foundation in a national survey [56]. We take the rarely or never categories combined as our dependent variable. We used the Warwick-Edinburgh Mental Wellbeing Scale (WEMWBS) as our other wellbeing outcome. This comprises 14 items covering positive affect and positive functioning over the past 2 weeks, with similar response categories to the loneliness question. Responses are summed to a scale from 14 to 70 with higher scores indicating higher wellbeing [57].

#### Physical activity

This was assessed using the short form International Physical Activity Questionnaire, IPAQ [58]. Respondents are asked on how many days in the past week, and for how long each day, they did vigorous activity, moderate activity and walking. From this scale we use two dependent variables: low activity (versus moderate or high); and total activity measured in MET-min per week. Respondents were also asked how much time they spent sitting down on a typical weekday; we used minutes sitting per day as a third dependent variable for physical activity.

#### Covariates

We included the following variables as potentially confounding factors in the analysis: gender; age group (< 40, 40–64, 65+); household type (adult, older person(s), family with children); employment status (working or full-time education, not-working, retired); educational qualifications (none; school or post-school); migrant status (British citizen, non-British), housing tenure (renter; owner); long-standing illness or disability (yes; no).

### Analysis

Analyses are based on a maximum of 3782 respondents (98.8%) after allowing for missing data on internet access or co-variates although further minor reductions in the number of respondents occur for particular outcome variables. Analyses of binary outcomes are based on logistic regression, with variables coded so that odds ratios represent the likelihood of a positive outcome, e.g. frequent social contact; not feeling lonely. To maximise statistical power, analyses of WEMWBS are based on least squares regression for a continuous outcome. Outcomes of interest were regressed, unadjusted and then adjusted for the covariates, on the four-level internet access variable. Analyses of loneliness and wellbeing were also repeated with additional adjustment for each of the social, amenities, and community outcomes in turn to explore potential mediation by these factors. Analyses were also repeated restricted to respondents aged 65+ with a binary internet access variable (any versus none) to allow for smaller numbers in this group.

## Results

A third of the sample (1236) said they did not use the internet. Nearly one-in-seven respondents (14.9%) accessed the internet by mobile phone only and a further third (35.4%) did so via their mobile phone and other means. By far the most common other means used by the latter group was home computer (94% of the group), followed by computer at work (22%) and access via other places (16%). Lastly, nearly one-in-six respondents (17.3%) exclusively accessed the internet by means other than mobile phone (89% by home computer, 4% by computer at work, and 10% via other places). In total therefore, half the sample (50.2%) accessed the internet via their mobile phone and nearly half did so via computer at home (48.6%).

Internet access or use was highest, at over 90%, among those aged under 40, those with dependent children and those in work or full-time education (Table 1). Conversely, internet access was lowest among households comprising older people, among whom only 28% accessed the internet, those with a long-standing illness or disability (46%) and those with no educational qualifications (52%). Reliance on mobile phones alone to access the internet was highest among those aged under 40 (24%), non-British (20%) and those out of work (20%). Internet access via computer at home was highest among those with dependent children (70%) and those in work or full-time education (71%). Although generally access to the internet via mobile phone was slightly higher than via home computer or elsewhere, there were some instances where the opposite was true: among older persons (those aged 65 or more, older person households and retired households), those with a long-standing illness or disability, and owner occupiers, more people accessed the internet via computer at home or elsewhere than via a mobile phone.

**Table 1:** Rates of internet access by respondent characteristics

	Does not use internet (*n* = 1236) %	Mobile access only (*n* = 565) %	Mobile plus other access (home or elsewhere) (*n* = 1346) %	Access internet at home or elsewhere (no mobile) (*n* = 657) %
Gender				
Males	34.0	14.9	33.9	17.3
Females	31.5	14.9	36.4	17.2
Age				
< 40	6.5	23.7	58.2	11..5
40–64	28.7	14.7	35.0	21.6
65+	72.2	3.8	7.0	16.9
Household type				
Family	8.2	18.5	58.7	14.6
Adult	25.1	18.5	37.7	18.7
Older	71.7	4.0	7.3	17.0
Employment status				
Working/education	7.5	17.5	59.7	15.3
Not working	35.5	19.7	26.2	18.5
Retired	67.2	5.3	8.7	18.8
Educational qualification				
None	47.7	11.7	23.6	17.1
Any	20.8	17.3	44.5	17.4
Migrant status				
British	35.0	14.3	33.0	17.8
Not British	11.4	20.0	55.5	13.1
Housing tenure				
Rented	33.5	16.0	34.5	16.1
Owned	27.9	9.4	39.7	23.0
Long standing illness or disability				
No	19.8	17.8	46.6	15.9
Yes	53.6	10.0	16.8	19.6

Results from fully adjusted analyses (Table 2) suggested that compared with respondents without internet access, those with access via mobile phone and other means (mostly computer at home) were more likely to have weekly contact with relatives (Odds ratio, OR (95% Confidence interval, CI): 1.23 (0.99, 1.54)) and friends (1.26 (1.00, 1.57)). Those accessing the internet by other means (mostly by computer at home), either with or without mobile phone access, were also more likely to have weekly contact with neighbours (OR (95% CI): 1.20 (0.94, 1.52) and 1.23 (0.96, 1.58) respectively). Respondents with internet access via mobile phone alone or via mobile phone and other means were also more likely to have financial social support available to them (1.23 (0.97, 1.56) and 1.23 (1.00, 1.52) respectively). Associations with other social contact and support outcomes were weaker and less consistent.

**Table 2:** Internet access and social contact and support

	Positive outcome (%)	OR (95% CI) unadjusted	OR (95% CI) adjusted for covariates^a^
At least weekly contact with relatives (*n* = 3758)			
No internet	68.7	1.00	1.00
Only mobile access	64.6	0.83 (0.67, 1.03)	1.04 (0.81, 1.33)
Mobile & other access	68.3	0.98 (0.83, 1.16)	1.23 (0.99, 1.54)
No mobile access	66.9	0.92 (0.75, 1.13)	1.01 (0.81, 1.25)
At least weekly contact with friends (*n* = 3767)			
No internet	63.3	1.00	1.00
Only mobile access	73.7	1.62 (1.30, 2.03)	1.14 (0.89, 1.47)
Mobile & other access	75.6	1.80 (1.52, 2.13)	1.26 (1.00, 1.57)
No mobile access	67.0	1.18 (0.96, 1.44)	0.99 (0.79, 1.22)
At least weekly contact with neighbours (*n* = 3763)			
No internet	76.2	1.00	1.00
Only mobile access	70.9	0.76 (0.60, 0.95)	1.02 (0.79, 1.33)
Mobile & other access	74.3	0.90 (0.75, 1.08)	1.20 (0.94, 1.52)
No mobile access	78.6	1.14 (0.91, 1.43)	1.23 (0.96, 1.58)
At least one person provides practical support (*n* = 3771)			
No internet	86.2	1.00	1.00
Only mobile access	82.8	0.77 (0.59, 1.01)	0.97 (0.71, 1.33)
Mobile & other access	84.8	0.90 (0.72, 1.12)	1.08 (0.81, 1.44)
No mobile access	86.8	1.06 (0.80, 1.39)	1.14 (0.85, 1.54)
At least one person provides financial support (n = 3763)			
No internet	55.5	1.00	1.00
Only mobile access	66.2	1.57 (1.27, 1.93)	1.23 (0.97, 1.56)
Mobile & other access	66.3	1.57 (1.34, 1.85)	1.23 (1.00, 1.52)
No mobile access	61.0	1.26 (1.04, 1.52)	1.12 (0.91, 1.37)
At least one person provides emotional support (n = 3758)			
No internet	83.7	1.00	1.00
Only mobile access	79.3	0.74 (0.58, 0.96)	0.78 (0.58, 1.04)
Mobile & other access	83.2	0.96 (0.78, 1.19)	0.95 (0.73, 1.25)
No mobile access	85.7	1.17 (0.90, 1.53)	1.17 (0.88, 1.56)

^a^Gender, age group, household type, employment status, educational qualification, migrant status, housing tenure, long standing illness or disability

There was a marked difference in the use of social amenities between those with and without internet access (Table 3). In adjusted analyses, those with internet access were more likely to have used social amenities in the past week than those who did not use the internet (OR (95% CI) for those accessing the internet by mobile phone alone: 1.25 (0.99, 1.59); for those accessing by mobile phone and other means: 1.32 (1.07, 1.62); and for those accessing exclusively by means other than a mobile phone 1.54 (1.25, 1.90)). Similarly, in adjusted analyses, those who accessed the internet via a mobile phone alone, or via a mobile phone and other means were approximately 60% more likely to have used shopping amenities in the past week (OR 1.58, 95% CI: 0.97, 2.58).

**Table 3:** Internet access and use of amenities

	Positive outcome (%)	OR (95% CI) unadjusted	OR (95% CI) adjusted for covariates^a^
Used any social amenities in the last week (*n* = 3782)			
No internet	38.5	1.00	1.00
Only mobile access	57.5	2.16 (1.77,2.65)	1.25 (0.99, 1.59)
Mobile & other access	62.8	2.70 (2.30, 3.16)	1.32 (1.07, 1.62)
No mobile access	56.9	2.11 (1.74, 2.56)	1.54 (1.25, 1.90)
Used any shopping amenities in the last week (*n* = 3782)			
No internet	89.1	1.00	1.00
Only mobile access	97.1	4.15 (2.45, 7.04)	1.62 (0.91, 2.89)
Mobile & other access	97.8	5.32 (3.55, 7.97)	1.58 (0.97, 2.58)
No mobile access	95.1	2.36 (1.59, 3.52)	1.36 (0.89, 2.08)

^a^Gender, age group, household type, employment status, educational qualification, migrant status, housing tenure, long standing illness or disability

Results from unadjusted models (Table 4) suggested that internet use was associated with a lower likelihood of knowing many people in the area, feeling part of the community, considering that neighbours looked out for each other, and anticipating that the community could improve things for itself. However, all associations were explained by adjustment for covariates. Internet use was not associated with a greater sense of political empowerment, with no difference in respondent’s assessment of their ability to influence decisions affecting the local area, or in their views on the responsiveness of service providers according to internet access.

**Table 4:** Internet access and sense of community

	Positive outcome (%)	OR (95% CI) unadjusted	OR (95% CI) adjusted for covariates^a^
Know most/many people in the neighbourhood (*n* = 3,777)			
No internet	48.5	1.00	1.00
Only mobile access	46.5	0.92 (0.76, 1.13)	1.18 (0.94, 1.50)
Mobile & other access	44.3	0.84 (0.72, 0.99)	1.11 (0.91, 1.37)
No mobile access	44.9	0.87 (0.72, 1.05)	0.93 (0.76, 1.14)
Feel a great deal part of the community (n = 3,747)			
No internet	43.2	1.00	1.00
Only mobile access	31.2	0.60 (0.48, 0.74)	0.87 (0.68, 1.11)
Mobile & other access	31.0	0.59 (0.50, 0.69)	0.87 (0.70, 1.08)
No mobile access	38.1	0.81 (0.67, 0.98)	0.94 (0.76, 1.16)
Neighbours look out for others a great deal (n = 3,697)			
No internet	27.2	1.00	1.00
Only mobile access	20.1	0.67 (0.53, 0.86)	0.86 (0.65, 1.14)
Mobile & other access	24.2	0.86 (0.72, 1.02)	1.13 (0.89, 1.43)
No mobile access	28.0	1.04 (0.84, 1.30)	1.10 (0.88, 1.39)
Community would intervene in case of harassment (n = 3,770)			
No internet	53.5	1.00	1.00
Only mobile access	51.9	0.94 (0.77, 1.15)	1.06 (0.84, 1.33)
Mobile & other access	54.5	1.04 (0.89, 1.22)	1.16 (0.94, 1.42)
No mobile access	54.9	1.06 (0.87, 1.28)	1.04 (0.84, 1.28)
Community would return lost purse/wallet (n = 3,769)			
No internet	29.1	1.00	1.00
Only mobile access	25.8	0.85 (0.68, 1.07)	1.02 (0.78, 1.32)
Mobile & other access	29.1	1.00 (0,85, 1.19)	1.18 (0.94, 1.48)
No mobile access	28.1	0.95 (0.77, 1.18)	0.99 (0.79, 1.24)
Agree can influence decisions (n = 3,768)			
No internet	45.7	1.00	1.00
Only mobile access	47.0	1.06 (0.86, 1.29)	1.17 (0.92, 1.47)
Mobile & other access	47.1	1.06 (0.91, 1.24)	1.16 (0.95, 1.43)
No mobile access	47.1	1.06 (0.87, 1.28)	1.00 (0.82, 1.23)
Agree people can improve things (n = 3,766)			
No internet	59.7	1.00	1.00
Only mobile access	53.7	0.78 (0.64, 0.96)	0.89 (0.70, 1.12)
Mobile & other access	55.2	0.83 (0.71, 0.97)	0.96 (0.78, 1.18)
No mobile access	61.4	1.07 (0.88, 1.30)	1.06 (0.86, 1.31)
Agree service providers are responsive (n = 3,765)			
No internet	61.9	1.00	1.00
Only mobile access	57.8	0.84 (0.69, 1.03)	1.00 (0.79, 1.26)
Mobile & other access	54.6	0.74 (0.63, 0.87)	0.91 (0.74, 1.12)
No mobile access	57.7	0.84 (0.69, 1.02)	0.92 (0.75, 1.13)

^a^Gender, age group, household type, employment status, educational qualification, migrant status, housing tenure, long standing illness or disability

Unadjusted results suggest that internet users were more likely to report being never or rarely lonely (Table 5). After adjustment for covariates, this was still true for those whoaccessed the internet via a mobile phone and other means (OR 1.46, 95% CI: 1.18, 1.82) or by other means alone (OR 1.66, 95% CI: 1.33, 2.07), but not for those who only accessed the internet via a mobile phone. Internet users also had higher mental wellbeing scores than non-users, this being true both before and after adjustment, particularly for those with internet access via mobile phone and other means (OR 2.62, 95% CI: 1.64, 3.61) and via other means alone (OR 2.16, 95% CI: 1.17, 3.16). Associations of lower loneliness and higher mental wellbeing among those with access to internet at home also remained unaltered when adjustments were made for the other social and community variables (results not shown).

**Table 5:** Internet access and wellbeing

	Positive outcome (%)	OR (95% CI) unadjusted	OR (95% CI) adjusted for covariates^a^
Rarely/never feel lonely (*N* = 3746)			
No internet	56.2	1.00	1.00
Only mobile access	58.5	1.10 (0.90, 1.34)	0.96 (0.76, 1.23)
Mobile & other access	71.5	1.96 (1.66, 2.31)	1.46 (1.18, 1.82)
No mobile access	69.6	1.78 (1.46, 2.19)	1.66 (1.33, 2.07)
	Mean (SD) score	Difference (95% CI) unadjusted	Difference (95% CI) adjusted for covariates^a^
WEMWBS score (*N* = 3704)			
No internet	47.4 (10.8)	0.00	0.00
Only mobile access	51.1 (11.3)	3.70 (2.62, 4.77)	1.35 (0.23, 2.46)
Mobile & other access	53.8 (10.4)	6.37 (5.53, 7.20)	2.62 (1.64, 3.61)
No mobile access	51.0 (10.4)	3.59 (2.56, 4.62)	2.16 (1.17, 3.16)

^a^Gender, age group, household type, employment status, educational qualification, migrant status, housing tenure, long standing illness or disability

Results for physical activity suggest that those with access to the internet were more active than those without (Table 6). After adjustment for factors such as age and disability, internet users were 30 % less likely to report low levels of physical activity, this being true both for all categories of internet access, for example those who accessed the internet via mobile phone alone (OR 0.66, 95% CI: 0.52, 0.85) and those who accessed the internet by means other than a mobile phone (OR 0.73, 95% CI: 0.59, 0.90). Further, those who accessed the internet via mobile phone had a markedly higher total level of physical activity across the week than non-users, especially those who accessed the internet exclusively via a mobile phone (an additional 478 (95% CI: 201, 755) MET-minutes per week). After adjustment for covariates, those who accessed the internet in any of the three categories reported sitting down for nearly 40 min fewer per day than those who did not use the internet (e.g. 38 (95% CI: 19, 58) fewer minutes per day among those accessing the internet via mobile phone or other means).

**Table 6:** Internet access and physical activity

	Positive outcome (%)	OR (95% CI) unadjusted	OR (95% CI) adjusted for covariates^a^
Low activity (versus moderate or high) (n = 3782)			
No internet	67.5	1.00	1.00
Only mobile access	39.6	0.32 (0.26, 0.39)	0.66 (0.52, 0.85)
Mobile & other access	33.9	0.25 (0.21, 0.29)	0.67 (0.54, 0.82)
No mobile access	49.2	0.47 (0.39, 0.57)	0.73 (0.59, 0.90)
	Mean (SD)	Difference (95% CI) unadjusted	Difference (95% CI) adjusted for covariates^a^
IPAQ score (total activity MET-min per week) (n = 3782)			
No internet	890 (1769)	0	0
Only mobile access	2329 (3059)	1439 (1176, 1701)	478 (201, 755)
Mobile & other access	2468 (3096)	1578 (1374, 1782)	264 (22, 506)
No mobile access	1692 (2551)	803 (553, 1053)	182 (−66, 430)
Minutes sitting per day (*n* = 3607)			
No internet	407 (230)	0	0
Only mobile access	311 (210)	−96 (−117, −76)	−39 (−61, −17)
Mobile & other access	290 (172)	−117 (− 133, −101)	−38 (−58, −19)
No mobile access	334 (199)	−73 (−93, −53)	−36 (−56, −17)

^a^Gender, age group, household type, employment status, educational qualification, migrant status, housing tenure, long standing illness or disability

The positive associations of outcomes with internet access observed in all respondents combined were also apparent in analyses restricted to the oldest age group and, in some cases, associations were stronger for those aged 65+. For example, after adjustment for covariates, compared with those without internet access, older people who used the internet were more likely to have weekly contact with family and friends (1.35 (0.98, 1.87)) and to use shopping amenities (1.89 (1.07, 3.36)). In addition, there were particularly marked associations of internet use with lack of loneliness (OR (95% CI): 1.72 (1.23, 2.39)) and positive wellbeing (difference (95% CI): 3.10 (1.78, 4.42)) in those aged 65 + .

## Discussion

We observed a number of social gains associated with the use of the internet among our respondents, particularly for those accessing the internet both via a mobile phone and elsewhere, including a higher likelihood of regular contact with relatives, friends and neighbours and a higher likelihood of available financial support (also true for those who only accessed the internet via a mobile phone). The finding on financial support was also reported in deprived communities in London, though the finding on neighbour contact elaborates on the earlier study, which reported that social ties mediated the relationship between internet access and wellbeing, though without separately reporting on neighbour contacts [47]. We did not find any consistent negative associations of internet access with social effects among our participants. Our findings are more consistent with the ‘social augmentation’ perspective on the internet [59] rather than the ‘social displacement’ perspective [3], namely that the internet provides additional avenues for social interaction and sources of social support and social identity, depending on a person’s prior level of social resources [60]. In particular, this ‘social compensation’ argument has previously been applied to those with ‘initially impoverished social resources’ or ‘stigmatised attributes’ [3, 60, 61], circumstances that are more likely to apply to people who live in deprived communities such as those studied here. Of course, it is possible that internet access reflects a prior or simultaneous higher level of social capital or other resources, thus confounding any ‘social augmentation’ findings; we cannot discount this, though we think it is less likely in our very deprived study communities than may generally be the case. The fact that we only found positive associations with social outcomes for those using the internet at home as well as by mobile phone, rather than for those whose main use of the internet is elsewhere or via a mobile phone alone may indicate that the two groups of users have different personalities, seek different things from on-line activity, or use the internet for different purposes. It is plausible that internet use at home by those with limited resources is more likely to generate proximate social contacts than distant ones and that nearby social contact is sought by those with home-based internet use.

Conversely, we did not find internet access to be associated with indicators of community cohesion or empowerment in the study communities. Based on our results, the argument that ‘online activities could increase people’s closeness to others and sense of belonging’ [3] would not appear to hold true in deprived areas at the scale of someone’s place of residence. In addition, our findings for deprived communities suggest caution in the optimism often expressed by governments and others about the benefits to citizens and democracies stemming from the internet. The internet is expected to generate a new generation of citizen associations with common agendas and interests as a result of the freedom from constraints of time, space and costs on participation [62]. Social media is said to have ‘returned power to citizens’ as it makes it easier for people to organise themselves and voice challenges, and also easier for government to collect citizens’ views and ideas, in these ways making governments more responsive and accountable [63]. We did not find any evidence that those living in deprived communities with access to the internet felt any more empowered than their co-residents without the internet, in terms of political decision-making, associational activity, or service responsiveness.

Our findings on the lack of association between internet access and community cohesion and empowerment may reflect a number of things. First, that internet access by either of the two main means (mobile phone and home computer) is at too low a level in such communities (just under half for each) for internet use to be effective in these regards, although which of the main means might be most effective for community cohesion and empowerment purposes is unknown and worthy of further investigation. Alternatively the results may be a function of what people are using the internet for, which we do not know for our study group. Lastly, there is a question about the extent of openness of the local authorities to the influence and involvement of citizens via the internet and, on the other side, a growing distrust by people regarding the way governments and others use the internet to influence citizens’ views [64]. The idea of developing a ‘digital government’ that will form part of the ‘sharing economy’ or ‘internet of things’ based on mutual trust and co-dependence between state and citizen (as for peer-to-peer goods and services), will require more change than simply placing more government services on-line [65]. Our results in this area are more indicative of a potential digital divide between middle-income and more affluent communities who can and do use the internet as a tool for greater empowerment, and poorer communities who do not or cannot; a disparity that is not fixable through a ‘technological approach’ alone [66].

The social benefits associated with internet use in our data extended to the use of social amenities and shops, suggesting that internet access does not crowd out activity in the real world and may actually help to facilitate it. The association with use of social amenities was stronger for those accessing the internet other than by mobile phone (mostly home computer), while the association with mobile phone access alone was stronger in the case of shops than social amenities. The latter finding is consistent with the use of smart phones (the main type of non-home internet access) for click-and-collect shopping, which has experienced rapid growth in the UK, dominated by clothes and footwear [67], and is a means by which those on constrained budgets (e.g. those who cannot afford home internet) can obtain goods and services at cheaper prices. This would suggest that internet access by any means may be of assistance to those in disadvantaged circumstances. The findings on greater use of amenities by those with internet access are similar to Scotland-wide results showing higher levels of internet access by those with ‘active lifestyles’ involving participation in cultural, sporting and voluntary activities and events [68]. However, the nature of the ‘active lifestyle’ lived by residents in deprived areas who use the internet may be different to the national typology, since levels of cultural engagement are lower in Scotland for those without post-school qualifications or who live in deprived areas [69].

The finding that internet access was associated with wellbeing advantages is important, not least because an earlier study which suggested that internet access was associated with psychological wellbeing moreso in disadvantaged groups called for replication of the finding in a larger study, which we have provided [47]. Furthermore, loneliness has previously been found to be associated with poor mental health, anxiety and depression [70], overeating and alcohol misuse [71], and damage to the cardiovascular system [72]. If internet access lowers the risk of loneliness, in accord with the argument that the internet is not a cause of loneliness [21], that is something worthy of further consideration by government and service providers. The association of internet access with higher mental wellbeing scores is also relevant to public policy in Scotland, where the government has included the improvement of mental wellbeing scores on the WEMWBS scale used here as part of its national performance framework [73]. However, the Government reports that there has been little progress on this national indicator since 2008, setting a threshold of +/− 0.4 as indicating “significant change” [74]. Thus, if internet services were to afford an improvement for those without current internet access of the order of magnitude found here (+ 2.42), this could amount to substantial progress on the national indicator for a proportion of the population. Although we cannot be certain, our results tend to indicate that home internet access (the main ‘other’ means of internet access recorded here) has stronger associations with wellbeing and social outcomes than access via a mobile phone alone. However, we have not recorded respondents’ incomes and it may be that those on the very lowest incomes tend to rely on internet access via mobile phone alone, so that weaker associations with social and wellbeing outcomes also mask effects of extreme poverty where deprived communities are concerned.

The wellbeing advantages of internet access were not countered in our study by physical activity disadvantages that have been identified elsewhere [34, 35]. Although one might reasonably expect that internet use outside the home would be associated with a certain level of physical activity, we also found a similar positive association with all means of internet use, and a negative association with sedentary behaviour. This suggests that conventional notions of what internet use or users are like may not apply to people living in deprived areas, not least because internet use is less often combined with car ownership in such places, with half the households in deprived areas in Scotland having no access to a car [75].

Across the range of outcomes examined, there were several positive associations with internet access for older people in particular, relating to social contact, use of amenities and health and wellbeing. The idea that internet access might assist older people with maintaining or renewing contact with friends is entirely plausible given that contacts can become distanced over time and people often seek to reconnect with old-friends later in life. The association between internet access and wellbeing for older adults is not surprising; others have argued that ‘active life engagement’ is important for health and wellbeing among older adults [76] and that the internet offers older people opportunities to learn new things, access services, take up hobbies and improve their quality of life [14]. Hence, it is concerning that we also found a large age-related digital divide, with the number of adults in deprived communities accessing the internet halving from middle age to retirement age. Overcoming such a divide for older people is likely to involve issues of digital literacy, technological competence and self-efficacy, and network access and associated costs [14, 77, 78].

The predominant means of accessing the internet other than by using a mobile phone is via a home computer; what is more, extending broadband access at home is a policy priority in the UK and Scotland. However, we found the prevalence of home internet access via a computer, laptop or tablet to be lower in deprived communities in Glasgow than the prevalence of home internet access reported for the most deprived quintile of neighbourhoods in Scotland in the same year, 49% versus 71%, respectively [75]. This may reflect the fact that deprived communities in Glasgow are even poorer than the most deprived fifth of areas across the country; our study communities were in the most deprived 15% of neighbourhoods at the commencement of our study, indicating that there may be significant differences in the experience of poverty between neighbourhoods, even at the lower end of the deprivation spectrum. Although cost is the most likely cause of lower internet access among those living in deprived areas, the issue of choices made by those on lower incomes is also relevant. Research on poverty and social exclusion in the UK has identified internet access as one of the few items (from a list of 76) that was not considered a necessity by most people, indeed even less so by respondents in Scotland than in the rest of the UK [79].

We also found inequalities in internet access within the deprived communities according to most of the dimensions we examined. There were particularly low rates of internet access by those who were older, retired, lacking educational qualifications, and with a long-standing illness or disability, and relatively high rates of home internet use by those with children, those in work or full-time education and non-British citizens. These patterns are similar to those reported previously in a study of deprived areas of London [47], with our finding for non-British citizens echoing the earlier finding that internet access was higher among more recent migrants, perhaps due to the need to keep in contact with relatives abroad in the earlier stages of migration and settlement.

We also found reduced internet access among those who rent (mostly social rent) rather than own their homes in deprived areas and the prevalence of home internet use (46%) was much lower than that reported for social renters across the country (62%) [75]. The importance of digital inclusion for social sector tenants is recognised by the sector and landlords themselves, particularly in relation to their tenants having fair access to services such as legal services and welfare benefits, much of which is being transferred online and will affect tenants’ welfare and ability to pay their rent [80]. While there has been a large investment programme in social housing over the past decade and a half to bring the housing stock up to the new Scottish Housing Quality Standard (SHQS), introduced in 2004 and revised by new guidance in 2011, advances in internet access still seem piecemeal. Whilst the revised SHQS guidance raised thermal insulation standards for reasons of comfort and related to wider government objectives around energy efficiency, the stipulations regarding home facilities and services remained framed around issues of health and safety, without any improvements to reflect qualify of life issues such as internet access [81].

Although the Scottish Government has lauded recent progress in increasing ‘broadband access at home’ among social sector tenants to 62%, this still means that nearly 40% of such tenants do not have home internet access. There is a paradox within the Government’s latest policy, which has key objectives of ‘digitising local services’ and ‘transforming the public sector’ [82] but fails to identify any means of overcoming the inequalities highlighted above, and appears unable to ensure universal access to the internet so that all citizens can operate effectively in an increasingly on-line world. Scotland’s Digital Strategy addresses issues of infrastructure (broadband in remote areas and superfast broadband) and skills (though mostly for employment purposes) and to a far lesser extent issues of the cost of accessing network services and purchasing technological devices with which to use the internet. However, a recent survey of disadvantaged citizens in Scotland found that the most important barriers to internet use concerned network costs [83].

### Strengths and limitations

The main strength of our study is the large sample of people from deprived communities, four times the size of a recent previous study of the same type of place [47]. Our results are based on a large number of comparisons and there is therefore potential for Type I errors. However, our focus in presenting these results is on identifying consistent associations across different measures of social contact, use of amenities, sense of community, wellbeing, loneliness, and physical activity rather than on isolated, conventionally “statistically significant”, associations. We also discuss our results in the context of existing evidence and identify where these are similar or differ. We believe the social integration results of this study are generalizable to other deprived communities in the UK, however the health and wellbeing results may not be reflected to the same extent elsewhere due to the relatively poor health of the population of Glasgow [84]. We have also controlled for many of the other factors that may have a strong effect upon wellbeing, including education, employment and long-standing illness. However, the cross-sectional nature of the data means that associations between internet access and wellbeing could run in either direction, although previous longitudinal research would support the notion that internet access is beneficial for social contact and wellbeing [62]. Although we allowed respondents to identify multiple ways in which they accessed the internet, we did not collect information on the duration or purpose of their internet use; these are important issues where further details for internet users in deprived communities would help us understand how and why the internet might be beneficial (or not) to those with fewer resources to spend on other forms of social interaction and activity.

## Conclusions

In the debate about the advantages and disadvantages of internet use, we have found a number of positive associations within deprived communities between internet access by adults and social integration and wellbeing outcomes and, further, we did not find any negative associations with internet access. Some of the associations were particularly evident for older people. Our findings provide direct support for one of the government’s claimed benefits of wider internet access [85], namely ‘keeping in touch’, and plausible grounds for supporting two others - online purchasing (via greater use of click and collect at shops) and accessing job vacancies (via a much higher rate of internet access by those in work living in deprived communities). More importantly, we found wellbeing benefits associated with internet access that did not appear to come at the cost of lower levels of physical activity, making the internet a potentially important contributor to the government’s strategy for tackling social isolation and loneliness in Scotland [86]. The question of what the appropriate policy response might be is not one we can completely answer. Some of our findings suggest that social and wellbeing outcomes are more strongly associated with internet access via home computer rather than via mobile phone (the two main means of accessing the internet), and it may be that internet access via computer and home broadband is more suitable for older people in particular. But for others, mobile phone access might be cheaper and more suitable; for example if younger adults are more residentially mobile or live in a shared flat. Thus, while internet access appears beneficial, the best means of providing it to lower income groups - for example by installing it into homes or by subsidising mobile phone data plans - is something requiring further investigation as policy options for different sub-groups.

However, the internet also has the capacity to solidify inequalities within society, with two issues in particular being highlighted by our study, both relating to greater official use of the internet. Health and care services are increasingly being organised and extended online, in order that people can access digital information, tools and services to maintain or improve their health [87]. Yet, in our study of deprived communities where health is often poorest, only a third of those with a long-standing illness or disability were able to access the internet at home, leaving two-thirds adrift from the convenience and immediacy of support via the internet. In addition, the move to digitise all public services and the interface between government and citizens risks disadvantaging poorer communities for whom we found no association between internet use and community cohesion or empowerment. There is, therefore, a risk that in the context of disadvantaged areas where access to the internet is lower and the ability to use the technology for collective organisation and empowerment less, the digitisation of the public sector could strengthen middle-class advantages in relation to public services and political decision-making.

## Data Availability

Requests to access the data can be made to the study’s data custodian Dr. Philip Mason: phil.mason@glasgow.ac.uk

## Abbreviations

CI: Confidence interval
GB: Gigabyte
IPAQ: International Physical Activity Questionnaire
Mb: Megabyte
OR: Odds ratio
SHQS: Scottish House Condition Survey
UK: United Kingdom
WEMWBS: Warwick-Edinburgh Mental Wellbeing Scale
